# Malaysian child infected with *Plasmodium vivax* via blood transfusion: a case report

**DOI:** 10.1186/1475-2875-12-308

**Published:** 2013-09-04

**Authors:** Claudia N Anthony, Yee-Ling Lau, Jia-Siang Sum, Mun-Yik Fong, Hany Ariffin, Wai-Linn Zaw, Indra Jeyajothi, Rohela Mahmud

**Affiliations:** 1 Department of Parasitology, Faculty of Medicine, University Malaya, 50603, Kuala Lumpur, Malaysia; 2 Department of Paediatrics, Faculty of Medicine, University Malaya, 50603, Kuala Lumpur, Malaysia; 3 University Malaya Medical Centre, Lembah Pantai, 59100, Kuala Lumpur, Malaysia

**Keywords:** *Plasmodium vivax*, Blood transfusion, Malaria transmission, Endemic regions, Microscopy, PCR

## Abstract

Malaria may be a serious complication of blood transfusion due to the asymptomatic persistence of parasites in some donors. This case report highlights the transfusion-transmitted malaria of *Plasmodium vivax* in a child diagnosed with germ cell tumour. This child had received blood transfusion from three donors and a week later started developing malaria like symptoms. Nested PCR and sequencing confirmed that one of the three donors was infected with *P. vivax* and this was transmitted to the 12-year-old child. To the best of the authors’ knowledge, this is the first reported transfusion-transmitted malaria case in Malaysia.

## Background

Although transfusion therapy aids in saving lives, blood can serve as a means to transmit infections, including parasitic infections [1]. Although the incidence of blood transfusion-transmitted parasitic infections (TTPI) is admittedly lower compared to viral and bacterial infections, it is important to understand that parasites can cause ailment, especially in immunocompromised individuals [2] Transfusion-transmitted malaria (TTM) occurs when the patient is infected by the same parasite that was present in the donor’s blood. TTM was first reported in 1911 [2-4]. While parasitic infections transmitted via accidental exposure to infected blood or blood transfusion may occasionally be difficult to diagnose in regions/areas where these infections are not endemic, such is usually not the case. TTM is in fact much harder to be identified in endemic countries as majority of donors may be potentially infected with malaria parasites [3]. Precise data on the incidence of TTM are under-reported in malaria-endemic areas, where recipients are known to have pre-existing infection. In non-endemic regions, the reported incidences range from zero to two cases per million donations [5]. As far as the authors are concerned, this is the first transfusion-acquired *Plasmodium vivax* malaria case reported in Malaysia.

## Case presentation

A 12-year-old Chinese male child diagnosed with intracranial malignant germ cell tumour received packed red cell transfusions at the University Malaya Medical Centre (UMMC), Malaysia in August 2012 for chemotherapy-induced anaemia. He received blood from three donors: a Myanmarese male and two Malaysian donors. A week later, upon returning to his home state, the patient started developing malaria-like symptoms. He sought consultation at the local hospital and was diagnosed with malaria following a blood test. He then returned to UMMC for further treatment. Blood samples from the patient and the three donors were sent to the Department of Parasitology, Faculty of Medicine, University of Malaya for further testing.

Giemsa-stained (5%) blood smear from the patient was prepared and examined under light microscope. Examination of the slide was carried out by a skilled microscopist and visualization of the blood smear showed a 0.03% parasitaemia *P. vivax* infection. Polymerase chain reaction (PCR) was carried out on the blood samples of the patient and the three donors to confirm the species of the malaria parasite. DNA was extracted from 100 μl of whole blood from the patient and the three donors using the DNeasy Blood & Tissue Kit (QIAGEN, Valencia, CA, USA). Nested PCR was performed based on the amplification of the small subunit ribosomal (SSU r) RNA gene developed previously [6]. The nest 1 reaction mixture of 25 μl contained 4 μl of DNA template, 5 pmol of nest 1 genus-specific primers (rPLU1: 5′-TCA AAG ATT AAG CCA TGC AAG TGA-3′and rPLU5: 5′-CCT GTT GTT GCC TTA AAC TCC-3′), 1× PCR buffer (35 mM Tris–HCl, pH 9.0, 3.5 mM MgCl2, 25 mM KCl, 0.01% gelatine), 0.25 M dNTP, 1 u *Taq* polymerase and 15.3 μl of nuclease free water. The nest 1 amplification conditions were as follows: 1) initial denaturation at 94°C for 4 min, 2) 35 cycles of denaturation at 94°C for 30 sec, annealing at 55°C for 1 min and extension at 72°C for 1 min, 3) final extension at 72°C for 10 min and a hold temperature of 4°C. Each of the nest 2 amplification mixture of 25 μl contained 4 μl of the nest 1 product and same amounts of buffer, dNTP, *Taq* polymerase and nuclease free water as in nest 1. The primer sets used in nest 2 were as follows: (rPLU3: 5′-TTT TTA TAA GGA TAA CTA CGG AAA AGC TGT-3′and rPLU4: 5′-TAC CCG TCA TAG CCA TGT TAG GCC AAT ACC-3′) for genus specific identification, FAL1: 5′-TTA AAC TGG TTT GGG AAA ACC AAA TAT ATT-3′ and FAL2: 5′-ACA CAA TGA ACT CAA TCA TGA CTA CCC GTC-3′ for *Plasmodium falciparum*, VIV1: 5′-CGC TTC TAG CTT AAT CCA CAT AAC TGA TAC-3′ and V1V2: 5′-ACT TCC AAG CCG AAG CAA AGA AAG TCC TTA-3′ for *P. vivax*, OVAL1: 5′-ATC TCT TTT GCT ATC TTT TTT TAG TAT TGG AGA- 3′ and OVAL2: 5′-GGA AAA GGA CAC ATT AAT TGT ATC CTA GTG-3′ for *Plasmodium ovale*, MAL1: 5′-ATA ACA TAG TTG TAC GTT AAG AAT AAC CGC-3′ and MAL2: 5′-AAA ATT CCC ATG CAT AAA AAA TTA TAC AAA- 3′ for *Plasmodium malariae*, Pmk8: 5′-GTT AGC GAG AGC CAC AAA AAA GCG AAT-3′ and Pmkr9: 5′-ACT CAA AGT AAC AAA ATC TTC CGT A-3′ for *Plasmodium knowlesi*. The nest 2 cycling conditions were identical to that of nest 1 except that an annealing temperature of 58°C was used for the species-specific primers. The PCR products were purified with QIAquick Gel Extraction Kit (QIAGEN, Valencia, CA, USA) and cloned into pGEM-T Vector Systems (Promega, Wisconsin, USA) prior to DNA sequencing. Ethical approval for this study was obtained from the Medical Ethics Committee of University Malaya Medical Centre (Reference no: 817.18).

Based on the PCR results, it was confirmed that the Myanmarese male donor was infected with *P. vivax* and the other two donors were found to be negative for malaria. The 26-year-old donor is from Sagaing Region, Myanmar and first came to Malaysia in 2008 and worked until June 2011 in Semenyih, Selangor. He then returned to Myanmar for four months and resumed working in Semenyih from October 2011 till today. The medical history of the donor did not suggest prior malaria infection nor was there other significant medical or surgical history. There was no prior history of receiving blood transfusion either. He first donated blood on 1 July, 2012 at a mobile blood campaign organized by UMMC in Kuala Lumpur. The donor was examined via microscopy during the donation but no parasites were detected.

**Figure 1:**
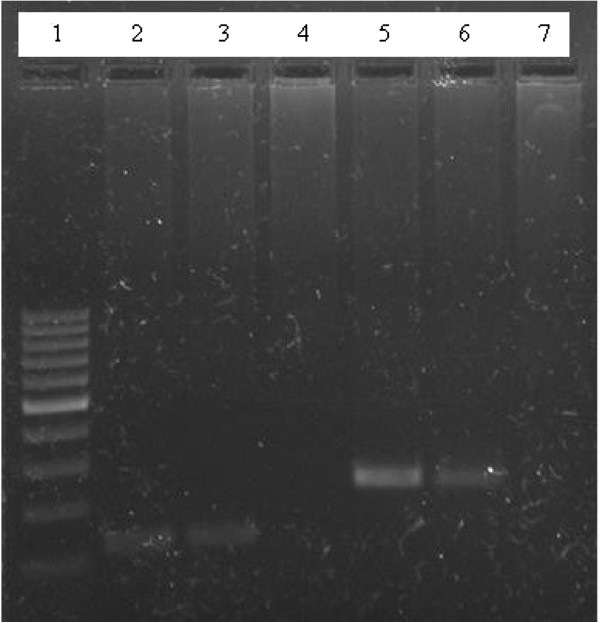
**Agarose gel image of patient and donor.** Lane 1 is the 100 bp marker. Lanes 2 and 3 are the amplification of both patient and donor DNA respectively with *P. vivax* species-specific primer. It is observed that both individuals show and amplification size on 120 bp, indicating *P. vivax* infection. Lane 4 is the negative control of the PCR run with the species-specific primer. Lanes 5 and 6 are the amplification of both patient and donor DNA respectively with the genus-specific primer. It is observed that they both indicate a size of 240 bp. Lane 7 is the negative control of the PCR run with the genus-specific primer.

**Figure 2:**
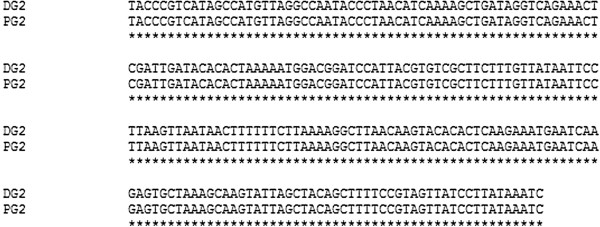
**Sequence alignment of patient and donor sequences.** It is observed that both patient and donor sequences are exactly the same. DG2 represents the genus sequence of the donor while PG2 represents the genus sequence of the patient.

An amplification size of 120 bp was observed for both the patient and the Myanmarese donor DNA samples (Figure 1). Sequence alignment studies using Clustal W 2.1 revealed that the two sequences from both individuals were exactly the same (Figure 2).

## Discussion

While malaria is endemic throughout most of the tropics, transmission of malaria is an unusual event in a non-endemic area. However, transmission is possible under certain circumstances such as (i) transmission by local competent mosquito vectors (ii) being infected by infective mosquitoes, that travelled aboard an airplane or within pieces of baggage to non-endemic areas (iii) nosocomial transmission, which involves a single incident of transmission to individual patients or hospital staff by blood transfusions, contingent needle-stick injuries or organ transplants [7,8]. In this report, transmission of malaria via blood transfusion between the patient and donor based on molecular investigations has been demonstrated.

In order for parasites to be transmitted by blood transfusion, parasites must circulate in the blood stream of donors, show certain physical characteristics and be able to survive conservation [2,9]. Such is the case with symptomatic malaria donors. In asymptomatic malaria donors however, the lack of clinical manifestations and subpatent level of parasites makes diagnosing the infection a difficulty [10]. Malaria presents itself as a febrile haemolytic disease that poses a greater threat to pregnant, asplenic or immunosuppressed patients [11]. It is a known fact that all four human malaria parasites (*P. falciparum*, *P. vivax*, *P. ovale,* and *P. malariae*) may be transfusion-transmitted [12]. After the first report in 1911, *P. vivax* was the predominant species until the 1950s when *P. malariae* replaced it as the most common causative agent [5]. In the 1970s, *P. vivax* was again the most common, followed by *P. malariae* and *P. falciparum.* An increasing number of transfusion-transmitted *P. falciparum* cases were observed at the same time with a high mortality rate [13]. In the past decade or so, several transfusion-transmitted malaria cases have been reported in Brazil [14,15], France [16], Republic of Korea [17], Colombia [18], USA [19] and UK [20].

Any blood component containing erythrocytes can harbour viable parasites. Whole blood and red blood cell (RBC) represent the most common sources of TTM. However, cases involving leukocytes, platelets, fresh frozen plasma, and frozen RBCs have been previously reported [5,14]. In both malaria-endemic and non-endemic countries, TTM can be a problem due to several characteristics of malaria infection: (a) partially immune individuals with low level parasitaemia remain asymptomatic and can qualify as blood donors; (b) *Plasmodium* is able to survive in blood stored at 4°C; and, (c) the sensitivity of the methods currently in use for detection of malaria is much lower than that required to detect level of parasitaemia capable of causing TTM [14,21]. In this case, it is possible that examination might have been performed by a non-expert who overlooked the donor as a potential threat due to sub-patent parasitaemia count. While blood safety remains an issue of great concern in transfusion medicine in all countries, it is of greater concern in developing countries where blood transfusion policies, trained personnel, appropriate infrastructure and financial resources are lacking or inadequate [1].

The donor, as previously mentioned, is from Sagaing Division, Myanmar. This location is situated in the north-west of Myanmar between Chin State on the west, Kachin State on the north-east, Shan State on the east and Magway Division and Mandalay Division on the south. Malaria is a major health issue in Myanmar, where resistant *P. falciparum* has already emerged [22]. Myanmar records an estimated 4.2 million cases annually and 69% of its population lives in malaria-endemic areas. Myanmar accounts for 75% of malaria cases and fatalities in the Mekong region and drug resistant malaria poses a greater threat [23]. In Sagaing Division, malaria cases are more prevalent in the months of June through November. These cases are predominantly due to *P. falciparum* but there are cases that are attributed to *P. vivax* as well [24]. In 2008, this division recorded an alarming 116,080 malaria cases, which placed it in second position from a list of 17 tested states/divisions [25]. It is an established fact that repeated exposure to malaria infection enables individuals to be protected against the disease as they would have progressively acquired antiparasite immunity [26]. Acquired semi-immunity could very well be the scenario with the donor as he comes from a region where transmission levels are high. Following an attack of malaria, a donor may remain infective for a long period of time, i.e., one to three years in *P. falciparum*, three to four years in *P. vivax* and for as long as 50 years in *P. malariae*[27].

Measures to monitor and assess transfusion transmission of infection, associating this to donor risk and evaluating possible threats to safety are pertinent [28]. There are two pivotal aspects to consider when taking into account malaria risk and transfusion: first, identification of malaria risk donors and second, managing the risk donors by either deferral or screening (including information of travel history and previous infection) [2,3]. A recent International Forum showed that in Europe and America, screening of donors by questionnaire is known to be the major deciding factor in prevention of transfusion-associated protozoan diseases [29]. It has been noted that most of the non-endemic countries follow the rule of donor deferral for 3 years after a donor has been infected with malaria [29]. While the development of donor-deferral guidelines that are appropriate to the country and to the donor population is of utmost importance to donor screening, one must bear in mind that complete prevention of TTM may not be possible [4]. In non-endemic countries, risk of transmission is minimized through donor deferral together with specific antimalarial antibody screening. In endemic countries however, more thorough donor questioning is required. This is coupled with knowledge of geographical distribution and seasonal variation to aid the identification of possibly infected donors [3]. A recent publication indicated that although malaria parasites are commonly found in the blood of donors in malaria-endemic areas, TTM does not occur frequently [30].

Before blood donation, the donor is required to fill out a consent and assessment form. Based on standard procedures, prior transfusion, a donor’s blood is subject to two tests. The first being the ABO blood group test and the second is a virology test, whereby the donor’s blood is screened for HIV, hepatitis B, hepatitis C and syphilis. In the rare event there are donors from malaria endemic countries, microscopic examination is performed to identify donors who may be potentially infected.

## Conclusion

Although no set of guidelines is flawless, appropriate deferral strategies coupled with appropriate laboratory screening will reduce the risk of TTM to a minimum. Donor selection is particularly difficult in healthy asymptomatic adults as they do not have fever and questionnaires do not identify such donors. However, with more rigorous questioning, there is a possibility of overcoming this problem. The *in vitro* processing of donor blood with antimalarials to kill parasites prior transfusion is also another measure to prevent TTM. In this study, the authors report a *P. vivax* transfusion-transmitted case which to the best of the authors’ knowledge is the first reported incident in Malaysia.

## Consent

Consent was granted by the patient for the publication of this case report.

## Competing interests

The authors declare that they have no competing interests.

## Authors’ contributions

CNA and LYL drafted and wrote the article, SJS performed the molecular diagnosis of the blood samples, FMY carried out the sequence analysis. RM performed the microscopy analysis, ZWL and IJ were the clinicians who dealt with the transfusion case. HA was the paediatrician attending to the patient. All authors read and approved the final manuscript.

## References

[B1] Ekwunife CA Ozumba NA Eneanya CI Nwaorgu OC Malaria infection among blood donors in Onitsha Urban, South Nigeria Sierra Leone J Biomed R 2011 3 21 26

[B2] Singh G Sehgal R Transfusion-transmitted parasitic infections Asian J Transfus Sci 2010 4 73 77 10.4103/0973-6247.67018 20859503 PMC2937300

[B3] Kitchen AD Chiodini PL Malaria and blood transfusion Vox Sang 2006 70 77 84 16430664 10.1111/j.1423-0410.2006.00733.x

[B4] Slinger R Giulivi A Bodie-Collins M Hindieh F St John R Sher G Goldman M Ricketts M Kain KC Transfusion-transmitted malaria in Canada CMAJ 2001 164 377 379 11232141 PMC80734

[B5] Seed CR Kitchen A Davis TME The current status and potential role of laboratory testing to prevent transfusion-transmitted malaria Transfus Med Rev 2005 19 229 240 10.1016/j.tmrv.2005.02.004 16010653

[B6] Singh B Lee KS Matusop A Radhakrishnan A Shamsul SS Cox-Singh J Thomas A Conway DJ A large focus of naturally acquired *Plasmodium knowlesi* infections in human beings Lancet 2004 363 1017 1024 10.1016/S0140-6736(04)15836-4 15051281

[B7] Zoller T Naucke TJ May J Hoffmeister B Flick H Williams CJ Frank C Bergmann F Suttorp N Mockenhaupt FP Malaria transmission in non-endemic areas: case report, review of the literature and implications for public health management Malar J 2009 8 71 10.1186/1475-2875-8-71 19379496 PMC2672952

[B8] Kirchgatter K Wunderlich G Branquinho MS Salles TM Lian YC Carneiro-Junior RA Di Santi SM Molecular typing of *Plasmodium falciparum* from Piemsa-stained blood smears confirms nosocomial malaria transmission Acta Trop 2002 84 199 203 10.1016/S0001-706X(02)00181-X 12443798

[B9] Garraud O Mechanisms of transfusion-linked parasite infection [in French] Transfus Clin Biol 2006 13 290 297 10.1016/j.tracli.2006.11.005 17188542

[B10] Laishram DD Sutton PL Nanda N Sharma VL Sobti RC Carlton JM Joshi H The complexities of malaria disease manifestations with a focus on asymptomatic malaria Malar J 2012 11 29 10.1186/1475-2875-11-29 22289302 PMC3342920

[B11] Marshall CS Unger KM Deckard-Janatpour K Risks of blood transfusion: challenges for the 21st century Semin Anesth 1998 17 195 207

[B12] Uneke CJ Ogbu O Nwojiji V Potential risk of induced malaria by blood transfusion in south-eastern Nigeria McGill J Med 2006 9 8 13 19529802 PMC2687905

[B13] Bruce-Chwatt LT Transfusion malaria revisited Trop Dis Bull 1982 79 827 840 6758249

[B14] Scuracchio P Vieira SD Dourado DA Bueno LM Colella R Ramos-Sanchez EM Lima GFMC Inoue J Sanchez MCA Di Santi SM Transfusion-transmitted malaria: case report of asymptomatic donor harbouring *Plasmodium malariae* Rev Inst Med Trop Sao Paulo 2011 53 55 59 10.1590/S0036-46652011000100010 21412621

[B15] Kirchgatter K Nogueira SL Padilha A Curado I Boulos M Di Santi SM Lethal malaria caused by *Plasmodium malariae* in an asplenic patient in Brazil BMJ 2005 331 7516 576b

[B16] Garraud O Assal A Pelletier B Danic B Kerleguer A David B Joussemet M de Micco P Overview of revised measures to prevent malaria transmission by blood transfusion in France Vox Sang 2008 95 226 231 10.1111/j.1423-0410.2008.01090.x 19121187

[B17] Lee YH Lee HK Choi KH Hah JO Lim SY Transfusion-induced malaria in a child after open heart surgery in Korea J Korean Med Sci 2001 16 789 791 11748364 10.3346/jkms.2001.16.6.789 PMC3054802

[B18] Echeverri D Barreto DK Osorio L Cortes A Martinez E A case report of transfusion-transmitted *Plasmodium vivax* malaria from an asymptomatic donor to a premature newborn [in Portuguese] Biomedica 2012 32 8 12 23235808 10.1590/S0120-41572012000500002

[B19] Reesink HW Panzer S Wendel S Levi JE Ullum H Ekblom-Kullberg S Seifried E Schmidt M Shinar E Prati D Berzuini A Ghosh S Flesland O Jeansson S Zhiburt E Piron M Sauleda S Ekermo B Eglin R Kitchen A Dodd RY Leiby DA Katz LM Kleinman S The use of malaria antibody tests in the prevention of transfusion-transmitted malaria Vox Sang 2010 98 468 478 10.1111/j.1423-0410.2009.01301.x 20136789

[B20] Kitchen A Mijovic A Hewitt P Transfusional transmitted malaria: current donor selection guidelines are not sufficient Vox Sang 2005 88 200 201 10.1111/j.1423-0410.2005.00610.x 15787731

[B21] Dubey A Elhence P Ghoshal U Verma A Seroprevalence of malaria in blood donors and multi-transfused patients in Northern India: relevance to prevention of transfusion-transmissible malaria Asian J Transfus Sci 2012 6 174 178 22988385 10.4103/0973-6247.98937 PMC3439759

[B22] Ejov MN Tun T Aung S Sein K Response of falciparum malaria to different antimalarials in Myanmar Bull World Health Organ 1999 77 244 249 10212515 PMC2557615

[B23] Regional cooperation is needed to fight malaria in Myanmar http://www.ausaid.gov.au/HotTopics/Pages/Display.aspx?QID=857

[B24] Malaria site: malaria in Asiahttp://www.malariasite.com/malaria/asia.htm

[B25] 2009-Asian collaborative training network for malaria http://www.actmalaria.net/IRW/IRW_Myanmar.pdf

[B26] Soe S Druilhe P The implications of naturally acquired immunity to malaria in Southeast Asia Trends Parasitol 2002 18 8 10 10.1016/S1471-4922(01)02138-9 11850004

[B27] Modi C Padalia U Patil RC A case study of possible relationship between post transfusion malaria and thalassaemia Asian J Exp Sci 2008 22 109 112

[B28] O’Brien SF Zou S Laperche S Brant LJ Seed CR Kleinman SH Surveillance of transfusion-transmissible infections: comparison of systems in five developed countries Transfus Med Rev 2012 26 38 57 10.1016/j.tmrv.2011.07.001 21944935 PMC7134890

[B29] Chauhan V Negi RC Verma B Thakur S Transfusion-transmitted malaria in a non-endemic area JAPI 2009 57 653 654 20214005

[B30] Owusu-Ofori AK Betson M Parry CM Stothard JR Bates I Transfusion-transmitted malaria in Ghana Clin Infect Dis 2013 56 1735 1741 10.1093/cid/cit130 23463635

